# Occlusal changes secondary to temporomandibular joint conditions: a critical review and implications for clinical practice

**DOI:** 10.1590/1678-775720150295

**Published:** 2016

**Authors:** Waleska CALDAS, Ana Cláudia de Castro Ferreira CONTI, Guilherme JANSON, CONTI Paulo César Rodrigues

**Affiliations:** 1 - Universidade de São Paulo, Faculdade de Odontologia de Bauru, Departamento de Odontopediatria, Ortodontia e Saúde Coletiva, Bauru, São Paulo, Brasil.; 2 - Universidade Norte do Paraná, Departamento de Ortodontia, Londrina, Paraná, Brasil.; 3 - Universidade de São Paulo, Faculdade de Odontologia de Bauru, Departamento de Prótese e Periodontia, Bauru, São Paulo, Brasil.

**Keywords:** Temporomandibular joint disorders, Malocclusion, Diagnosis

## Abstract

The relationship between Temporomandibular Disorders (TMD) and malocclusion is an extremely critical issue in dentistry. Contrary to the old concept that malocclusion causes TMD, occlusal changes, especially those observed as sudden, may be secondary and reflect joint or muscle disorders due to the obvious connection between these structures and the dental occlusion.

**Objectives:**

The aim of this article is to present the most commonly occlusal changes secondary to TMD.

**Methods:**

The clinical presentation of these conditions is discussed. Details regarding diagnosis, treatment, and follow-up of patients presenting TMD prior or during treatment are also presented.

**Conclusions:**

All plans for irreversible therapy should be preceded by a meticulous analysis of TMD signs and symptoms in such a way that patients are not submitted to irreversible treatment, based on an untrue occlusal relationship, secondary to articular and/or muscular disorders. When present, TMD symptoms must always be controlled to reestablish a “normal” occlusion and allow proper treatment strategy.

## INTRODUCTION

The relationship between Temporomandibular Disorders (TMD) and malocclusion is an extremely critical issue in dentistry. In the 1980s, a lawsuit declared that orthodontic treatment was the main cause of TMD^23^. Since then, a significant number of studies have been conducted to investigate this association.

In the past, studies have suggested that malocclusion and occlusal interferences were main factors in TMD development, thus, validating irreversible occlusal therapies as the definitive treatment of the disorder^9,25^. Based on that, occlusal adjustments, full mouth rehabilitation and orthodontic treatment became very popular as the treatment of choice for TMD.

However, most recent studies have shown no difference in relation to signs and symptoms of TMD among subjects with malocclusion and those with normal occlusion^3,20^ as well as between orthodontically treated and non-treated individuals^3,13^.

In earlier 1990s, well conducted studies have demonstrated that some occlusal/skeletal factors, such as anterior open bite, unilateral posterior crossbite, overjet greater than 6-7 mm, absence of five or more posterior teeth, and centric relation (CR) to maximum intercuspation (MI) discrepancy greater than 2 mm could be considered occlusal risk factors for TMD^14,16,24^. However, most of the people presenting these alterations have never experience any TMD symptoms. An appropriate adaptation capacity is probably able to compensate possible small alterations in function, created by the presence of the malocclusion^18^.

Most of these findings come from cross-sectional studies and would reflect a possible association between these variables, which, although valid, do not allow a temporal characterization of the variables. In other words, it cannot be established which variable (occlusal changes and TMD) developed first. To establish a cause-effect relationship, prospective longitudinal studies with large and representative samples are needed, but are not yet available.

Thus, contrary to the old concept that malocclusion causes TMD, it may be that, due to the obvious connection between these structures and the dental occlusion, occlusal changes, especially those suddenly observed, may be secondary and reflect joint (Temporomandibular Joint - TMJ) or muscle disorders^15^.

An acute malocclusion refers to any sudden change in the occlusal relationship that has been developed by a disorder^24^. This may be a momentary or prolonged condition^29^.

Some of the most commonly TMJ conditions associated with occlusal changes are joint effusion due to inflammation^15^ and condylar degeneration processes associated or not with systemic problems^2^.

The aim of this article is to present some conditions of occlusal changes, secondary to temporomandibular joint conditions, introducing the dental professional to the great importance of their recognition and of the evaluation of signs and symptoms of TMD prior to treatment planning.

## OCCLUSAL CHANGES SECONDARY TO TEMPOROMANDIBULAR DISORDERS

The development of a malocclusion, associated with signs and symptoms of TMD, is an unusual complaint in an orthodontic clinical practice^18^. When an occlusal alteration is caused by TMD, the resulting mandibular position and occlusal relationship depends on the TMJ structures and/or muscles involved^24^. Patients can demonstrate any of a number of clinical conditions that interfere with their comfort and ability to function^5^.

It is, therefore, essential in this scenario that clinicians are qualified to recognize these conditions before beginning any treatment planning, in such a way that patients are not submitted to irreversible treatment (orthodontic treatment, occlusal adjustment, prosthetic rehabilitation, orthognathic surgery), based on an unstable occlusal relationship, produced by articular and/or muscular disturbances. The implementation of immediate treatment would not only bring no benefit to the patient’s symptoms improvement, but it could also aggravate TMD severity.

The clinical presentation of most commonly occlusal changes secondary to signs and symptoms of TMD is further discussed.

### Anterior open bite (AOB)

An anterior open bite is a very common finding in patients with TMJ degenerative diseases.

Temporomandibular Joint osteoarthritis associated with functional overloading can lead to joint tissues collapse. If the joint collapse occurs in both TMJs, condylar resorption causes morphologic breakdown of the TMJs and a subsequent decrease in ramus height, which results in progressive mandibular retrusion with anterior open bite. This malocclusion is called “acquired open bite associated with TMJ osteoarthritis”^26^.

Temporomandibular Joint degenerative diseases may be consequent to systemic conditions. It has been demonstrated that rheumatoid arthritis, among other reumathological conditions, can affect the TMJ^7,21^. Individuals with these conditions have less occlusal support, more occlusal interferences, greater discrepancy between Centric Relation and Maximum Intercuspation, and decreased vertical overbite. Anterior open bite is, perhaps, the most common clinical finding in this situation, correlated to reduced maximum mouth opening capacity^26^.

In patients with such conditions, anterior teeth show wear facets and the absence of mamelons on the incisal edges, indicating that non-contacting teeth of these patients used to be in contact before^2^.

Patients with TMJ osteoarthritis generally reports TMJ and muscle pain, aggravated by jaw movement, and joint crepitation^12,18^, confirmed by clinical palpation and inspection.

The diagnosis is usually confirmed by appropriate TMJ images and blood tests (rheumatoid factor, erythrocyte sedimentation, antinuclear antibodies), especially when systemic conditions are present. Whenever condylar resorption is detected, it is essential to define the stage of resorption to investigate whether the destructive process is active or already came to a “burn out” phase. Tests, such as computed tomography (CT), magnetic resonance image (MRI) and bone scintigraphy are useful tools for that purpose. Cone-beam CT is an excellent option because of its capacity to adequately detect bone changes, a usual finding in this scenario. The MRI, on the other hand, allows visualization of the disc position and articular cartilage alterations^19^.

The frequent radiographic findings comprise erosion and flattening of the articular surface of the condyle and articular eminence, osteophytes, articular cysts, and loss of the joint space^18^.

While the TMJ osteoarthritis can produce relatively minor open bite changes fairly slowly, the “idiopathic condylar resorption”, a condition that preferentially affects women, and is influenced by hormonal changes and external triggers, such as orthognathic surgery or other traumas^7^, is, in comparison, much more aggressive and can lead to severe open bite in a relatively short time^21^.

**Figure 1:**
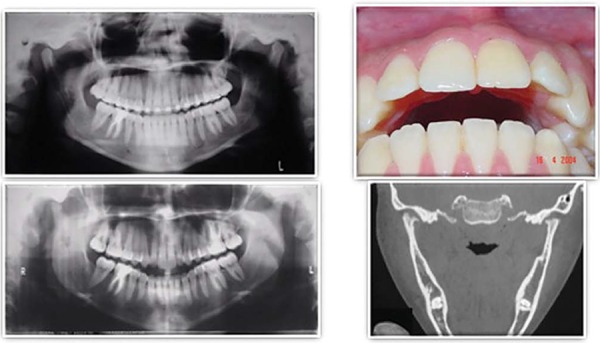
Anterior open bite secondary to rheumatoid arthritis radiographic and clinical images

In Figure 1, despite all the well-known limitations of the panoramic radiograph technique to analyze condyle’s shape, it is possible to note the development of condylar resorption in a 16-year-old female patient, with past medical history of rheumatoid arthritis, presented to the dental office with a chief complaint of TMJ pain, associated with a severe occlusal relationship change (anterior open bite).

The computed tomography (Figure 1) clearly shows the advanced degree of condylar resorption, associated with the presence of osteophytes and condylar erosions.

Severe anterior open bite, with teeth contact only in the posterior region (Figure 1), is present, resulting in functional problems such as chewing and speaking difficulties as well as intense esthetic deficit.

Although not a common complaint^30^, progressive anterior open bite, as presented here, should always be considered as part of the TMJ degenerative disease process.

### Unilateral posterior open bite

#### Associated with unilateral condylar resorption

When condylar resorption occurs unilaterally, an intrusion of the condyle, associated with mandibular shift to the affected side is a common finding. The result is an anterior open bite associated with a posterior open bite on the contralateral side, with occlusal contact occurring only on the posterior region of the affected side^28^.

**Figure 2:**
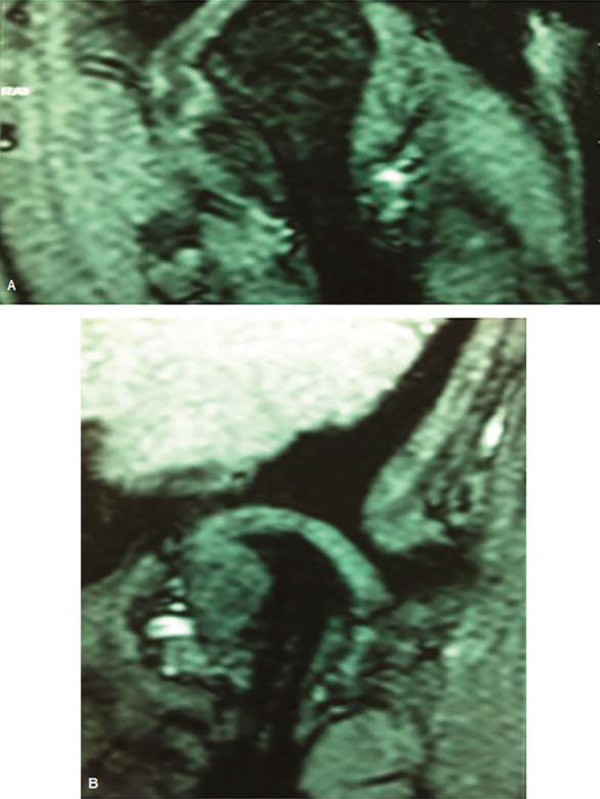
Magnetic resonance image evidencing integrity of condyle cortical on the right side (A) and a high degree of condylar resorption on the left side (B) of a patient presenting posterior open bite on the unaffected side

**Figure 3:**
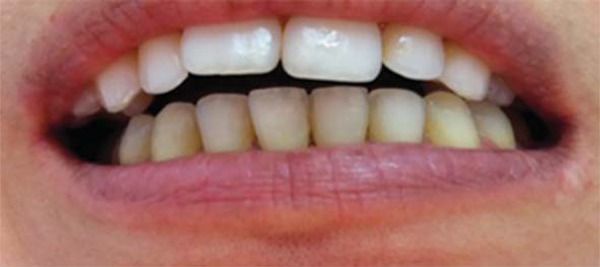
Posterior open bite on the right side secondary to resorption of the opposite side condyle

In Figure 2, based on the MRI, it is possible to note the integrity of condyle cortical on the right side and a high degree of condylar resorption on the left side in a 39-year-old patient, previously submitted to orthognathic surgery. Left TMJ pain and sudden posterior open bite on the right side were initial complaints (Figure 3).

#### Associated with unilateral joint effusion

**Figure 4:**
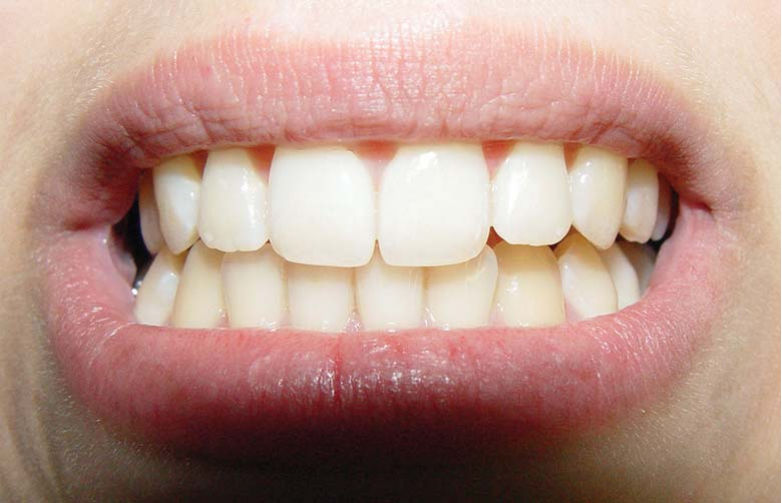
Joint effusion on the left temporomandibular joint resulting in a posterior open bite on the affected side

**Figure 5:**
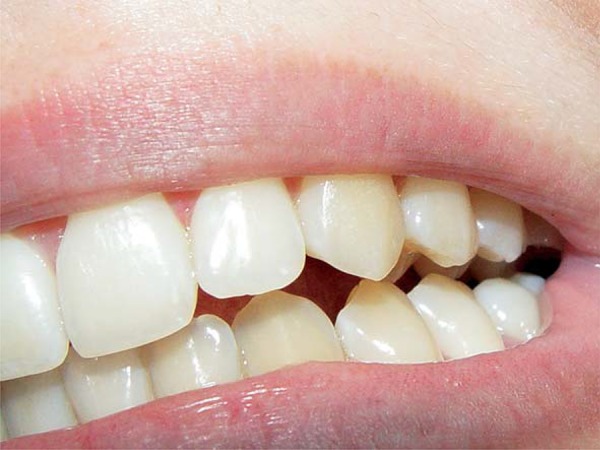
Mandible shift accompanied by a lower midline deviation to the right side secondary to joint effusion on the opposite temporomandibular joint

Certain intracapsular inflammatory disorders can also lead to occlusal alterations. TMJ retrodiscal tissues are highly vascularized and innervated, and therefore susceptible to the installation of inflammatory processes (retrodiscitis)^24^, which can lead to joint effusion. The joint effusion results from accumulation of inflammatory liquid that prevents a perfect fitting of the TMJ condyle into the mandibular fossa. The outcome of this condition is a sudden development of ipsilateral posterior open bite, and a strong contact on the canine region on the opposite side^24^. This mandible shift is accompanied by a lower midline deviation (Figures 4 and 5).

Patients present ipsilateral pain in the preauricular region. It is also important to be aware of more severe disorders that can cause similar symptoms and develop malocclusions such as TMJ tumors or ear infections^29^.

Additionally, longitudinal studies and regression analysis of data would be useful for establishing which occlusal changes are due to TMD, which to our knowledge are still not available in the literature.

## Initial consultation and the presence of TMD signs and symptoms

Occlusal relationship is frequently disturbed by TMD manifestations, as previously mentioned, and the dental professionals must always be aware of the presence of such signs and symptoms prior to any irreversible procedure.

**Figure 6:**
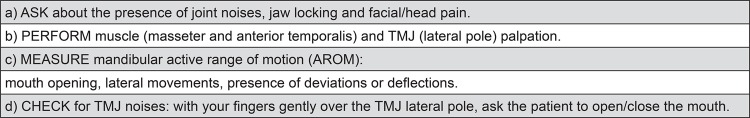
Assessment of signs and symptoms of TMD

There are different protocols for the assessment of signs and symptoms of TMD well established in the literature, such as the Research Diagnostic Criteria (RDC/TMD)^6^ or the Helkimo indices^8^. Although these protocols involve parameters that are very important for the diagnostic and qualification of the disorders by specialists in TMD and researches, the assessment of possible signs and symptoms of TMD can be performed in a relatively short time, with no significant increase in the time taken to complete the initial examination. Suggestions for these procedures are shown in Figure 6. These procedures allow the clinician to identify the presence of the disorder and refer the patient to a TMD specialist before starting any irreversible treatment. Before treatment, patients should always be asked about history of symptoms of TMD such as TMJ noises, jaw locking, and pain in the region of facial muscles, joints or temple area. Clinically, it is suggested to inspect for tenderness to palpation on the region of the masseter, anterior temporalis muscles, and in TMJ region. Assessment of mandibular active range of motion (AROM), as well as inspection of joint noises, should also be performed.

**Figure 7:**
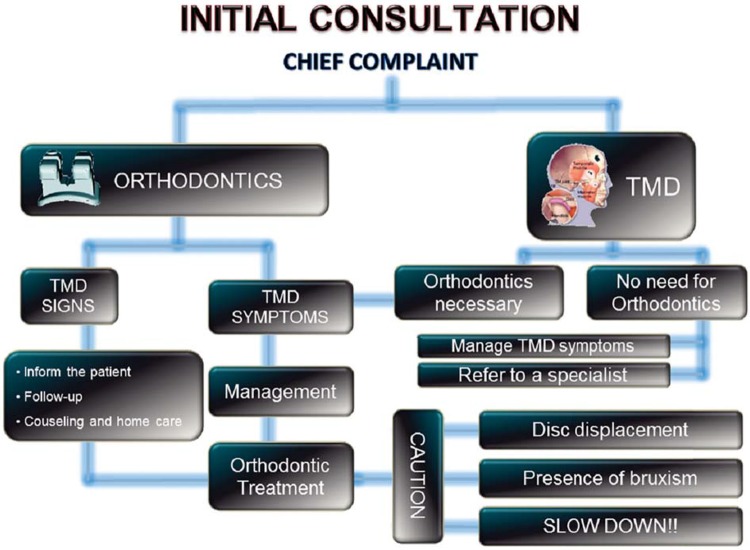
Protocol of management of patients presenting signs and symptoms of temporomandibular disorder before orthodontic treatment

An algorithm is suggested (Figure 7) to assist clinicians on how to proceed when dealing with these situations. This algorithm is based on the main reason why patients come to the dental office: for orthodontic treatment (functional/esthetic complaints) or for TMJ/masticatory muscles pain and dysfunction.

**Figure 8:**
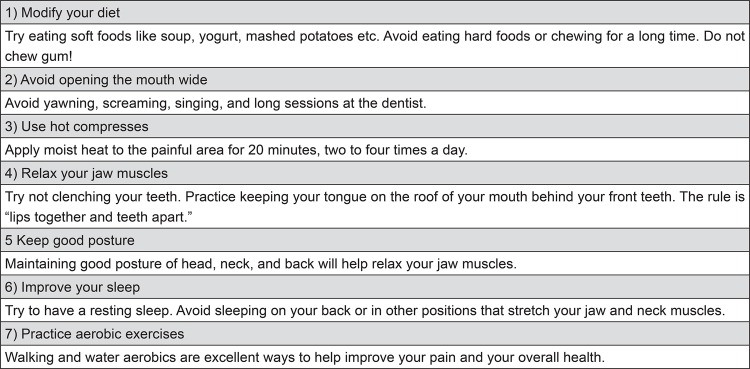
Counseling and home self-care guidelines for the TMD patient

According to the suggested protocol, when patients come for orthodontic treatment and signs of TMD (not painful symptoms) are detected at baseline, it is essential that the patient is informed prior to the beginning of treatment. This is important because, in case of progress of signs to symptoms during the course of treatment, patients could consider that the treatment was the cause of the disorder. It is important at this time that the practitioner is able to detect any contributing factor such as sleep or daytime bruxism/clenching, nail biting, chewing gum, deleterious sleep position, among others. A complete counseling and behavioral modification strategy should be adopted to avoid the progression of signs as suggested by the protocol of the American Academy of Orofacial Pain (AAOP)^4^. A few basic orientations that can be given to patients with TMD signs are shown in Figure 8.

Asymptomatic joint clicking, one the most common signs of TMD^11,22^, is one example of this situation. It is a condition that usually do not require treatment^27^, but can progress to symptoms^1^ if the patient has uncontrolled and persistent deleterious parafunctional habits.

In these cases, orthodontic treatment can be initiated. The patient should be frequently reassessed regarding the detected signs and repeatedly have general orientation on how to avoid the progression of the disorder. Additionally, patients may not report pain on initial stages of degenerative joint diseases. When facing an occlusal change, as stated before, dentists should consider conducting further investigations on TMJ (including images).

On the other hand, if a patient is looking for orthodontic treatment, but also has TMD and this is the chief complaint, it is very important that orthodontic treatment is not initiated. When present, symptoms must be properly managed before the initiation of orthodontic therapy. This is because, as previously stated, many TMD manifestations can result in an unstable occlusal relationship, interfering with a correct treatment planning.

When any signs or symptoms of TMD are observed, the patient should be referred to a TMD specialist (or a dentist with specific training on TMD) for further evaluation, diagnostic, and management. These professionals are prepared to conduct a differential diagnostic and to follow the patient during the orthodontic treatment or any occlusal therapy.

Therapy usually follows a conservative treatment protocol^17^, including pharmacotherapy, behavioral counseling (Figure 8), home exercises, physical therapy, and/or intraoral appliances^18^. To date, evidence based dentistry (EBD) does not support therapies that promote complex and irreversible occlusal changes such as occlusal adjustment, orthodontic treatment, functional orthopedics, orthognathic surgery or prosthetic oral rehabilitation for the treatment of TMD^10^. This discussion, however, is not within the scope of this article.

Once the pain has been resolved and the condition is stable over a reasonable amount of time, initiation of orthodontic therapy may be considered^18^. Treatment plan must consider possible vulnerabilities of the TMD patient such as asymptomatic anteriorly disc displacement or remaining parafunctional habits.

## Developing signs and symptoms of TMD during Orthodontic treatment

TMD signs and symptoms are particularly fluctuating, and can appear during the course of orthodontic treatment. Regular orthodontic treatment is done on adolescents, a stage when there is a natural increase of many contributing factors for TMD such as trauma, habits, emotional stressors, etc. Hence, it is not uncommon that, regardless of the orthodontic modalities, individuals develop transient signs/symptoms at that life stage. It is important that the orthodontist notify the patient that these problems are highly prevalent in the general population and that the etiology is multifactorial. Therefore, it is not possible to establish a correlation with the orthodontic therapy^18^.

Usually, the use of basic pharmacotherapy, such as muscle relaxants ad non-steroidal anti-inflammatory (NSAID) medications, associated with counseling, physical therapy and behavioral modification would be sufficient to control these signs and symptoms. However, if there is an indication for the use of intraoral splints as part of the management strategy, the orthodontic therapy must be discontinued and restarted only after all symptoms were properly addressed.

**Figure 9:**
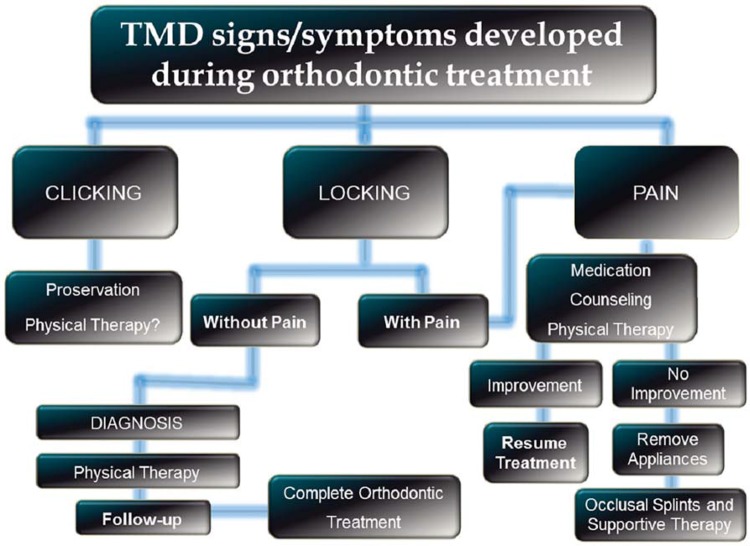
Protocol of management of patients presenting signs and symptoms of temporomandibular disorder during orthodontic treatment

A protocol on how to manage patients presenting signs and symptoms of TMD during orthodontic treatment is shown in Figure 9.

## CONCLUSIONS

Based on the fact that there is an evident connection between TMJ, masticatory muscles, and the dental occlusion, occlusal changes may reflect the presence of TMD. Therefore, all plans for irreversible therapy, such as orthodontics or prosthetic rehabilitation, should be preceded by a meticulous analysis of TMD signs and symptoms. When present, TMD symptoms must always be controlled to reestablish a normal occlusion and allow proper treatment strategy.

## References

[1] Bonjardim LR, Gavião MB, Pereira LJ, Castelo PM, Garcia RC (2005). Signs and symptoms of temporomandibular disorders in adolescents. Braz Oral Res.

[2] Chen YJ, Shih TT, Wang JS, Wang HY, Shiau YY (2005). Magnetic resonance images of the temporomandibular joints of patients with acquired open bite. Oral Surg Oral Med Oral Pathol Oral Radiol Endod.

[3] Conti A, Freitas M, Conti P, Henriques J, Janson G (2003). Relationship between signs and symptoms of temporomandibular disorders and orthodontic treatment: a cross-sectional study. Angle Orthod.

[4] Leew R, Klasser GD (2013). Orofacial pain: guidelines for assessment, diagnosis and management.

[5] Dupont JS (2006). Acute malocclusion. Gen Dent.

[6] Dworkin SF, LeResche L (1992). Research diagnostic criteria for temporomandibular disorders: review, criteria, examinations and specifications, critique. J Craniomandib Disord.

[7] Gunson MJ, Arnett GW, Milam SB (2012). Pathophysiology and pharmacologic control of osseous mandibular condylar resorption. J Oral Maxillofac Surg.

[8] Helkimo M (1974). Studies on function and dysfunction of the masticatory system. II. Index for anamnestic and clinical dysfunction and occlusal state. Sven Tandlak Tidskr.

[9] Henrikson T, Nilner M (2003). Temporomandibular disorders, occlusion and orthodontic treatment. J Orthod.

[10] Koh H, Robinson PG (2004). Occlusal adjustment for treating and preventing temporomandibular joint disorders. J Oral Rehabil.

[11] Kohler AA, Helkimo AN, Magnusson T, Hugoson A (2009). Prevalence of symptoms and signs indicative of temporomandibular disorders in children and adolescents. A cross-sectional epidemiological investigation covering two decades. Eur Arch Paediatr Dent.

[12] Kurt H, Oztaş E, Gençel B, Taşan DA, Oztaş D (2011). An adult case of temporomandibular joint osteoarthritis treated with splint therapy and the subsequent orthodontic occlusal reconstruction. Contemporary Clinical Dentistry.

[13] Macfarlane TV, Kenealy P, Kingdon HA, Mohlin BO, Pilley JR, Richmond S (2009). Twenty-year cohort study of health gain from orthodontic treatment: temporomandibular disorders. Am J Orthod Dentofacial Orthop.

[14] Magnusson T, Egermarki I, Carlsson GE (2005). A prospective investigation over two decades on signs and symptoms of temporomandibular disorders and associated variables. A final summary. Acta Odontol Scand.

[15] Marinho LH, McLoughlin PM (1994). Lateral open bite resulting from acute temporomandibular joint effusion. Br J Oral Maxillofac Surg.

[16] McNamara JA, Seligman DA, Okeson JP (1995). Occlusion, orthodontic treatment, and temporomandibular disorders: a review. J Orofac Pain.

[17] Michelotti A, Wijer A, Steenks M, Farella M (2005). Home-exercise regimes for the management of non-specific temporomandibular disorders. J Oral Rehabil.

[18] Michelotti A, Iodice G (2010). The role of orthodontics in temporomandibular disorders. J Oral Rehabil.

[19] Miller E, Uleryk E, Doria AS (2009). Evidence-based outcomes of studies addressing diagnostic accuracy of MRI of juvenile idiopathic arthritis. AJR Am J Roentgenol.

[20] Mohlin B, Axelsson S, Paulin G, Pietilä T, Bondemark L, Brattström V (2007). TMD in relation to malocclusion and orthodontic treatment. Angle Orthod.

[21] Müller L, Kellenberger CJ, Cannizzaro E, Ettlin D, Schraner T, Bolt IB (2009). Early diagnosis of temporomandibular joint involvement in juvenile idiopathic arthritis: a pilot study comparing clinical examination and ultrasound to magnetic resonance imaging. Rheumatology (Oxford).

[22] Nydell A, Helkimo M, Koch G (1994). Craniomandibular disorders in children - a critical review of the literature. Swed Dent J.

[23] Pollack B (1988). Michigan jury awards +850,000 in ortho case: a tempest in a teapot. Am J Orthod Dentofacial Orthop.

[24] Pullinger AG, Seligman DA, Gornbein JA (1993). A multiple logistic regression analysis of the risk and relative odds of temporomandibular disorders as a function of common occlusal features. J Dent Res.

[25] Sonnesen L, Bakke M, Solow B (1998). Malocclusion traits and symptoms and signs of temporomandibular disorders in children with severe malocclusion. Eur J Orthod.

[26] Tanaka E, Yamano E, Inubushi T, Kuroda S (2012). Management of acquired open bite associated with temporomandibular joint osteoarthritis using miniscrew anchorage. Korean J Orthod.

[27] Torii K (2011). Longitudinal course of temporomandibular joint sounds in Japanese children and adolescents. Head Face Med.

[28] Wolford LM, Cardenas L (1999). Idiopathic condylar resorption: diagnosis, treatment protocol, and outcomes. Am J Orthod Dentofacial Orthop.

[29] Wright E (2009). Manual of temporomandibular disorders.

[30] Yamada K, Satou Y, Hanada K, Hayashi T, Ito J (2001). A case of anterior open bite developing during adolescence. J Orthod.

